# Environmental heterogeneity and commodity sharing in smallholder agroecosystems

**DOI:** 10.1371/journal.pone.0228021

**Published:** 2020-01-29

**Authors:** Stacey A. Giroux, Paul McCord, Sara Lopus, Drew Gower, Jampel Dell’Angelo, Stephanie Dickinson, Xiwei Chen, Kelly K. Caylor, Tom P. Evans

**Affiliations:** 1 Ostrom Workshop, Indiana University, Bloomington, Indiana, United States of America; 2 Department of Anthropology, Indiana University, Bloomington, Indiana, United States of America; 3 Center for Systems Integration and Sustainability, Department of Fisheries and Wildlife, Michigan State University, East Lansing, Michigan, United States of America; 4 Department of Social Sciences, California Polytechnic State University, San Luis Obispo, California, United States of America; 5 Department of Civil and Environmental Engineering, Princeton University, Princeton, New Jersey, United States of America; 6 Department of Environmental Policy Analysis, Institute for Environmental Studies, VU University Amsterdam, Amsterdam, The Netherlands; 7 School of Public Health, Epidemiology and Biostatistics, Indiana University, Bloomington, Indiana, United States of America; 8 Department of Geography, University of California, Santa Barbara, California, United States of America; 9 Bren School of Environmental Science and Management, University of California, Santa Barbara, California, United States of America; 10 School of Geography and Development, University of Arizona, Tucson, Arizona, United States of America; University of Leeds, UNITED KINGDOM

## Abstract

Smallholder farmers undertake a number of strategies to cope with climate shocks in a community. The sharing of resources across households constitutes one coping mechanism when environmental shocks differentially impact households. This paper investigates commodity sharing dynamics among households in eight communities in an environmentally heterogeneous highland-lowland area in central Kenya. We use survey data and meteorological data to test whether commodity sharing, measured at the household level by net inflow of commodities, varies across a regional precipitation gradient, and we reveal how sharing fluctuates with rainfall over the course of a year. We find both precipitation and income to be significant predictors of households’ net value of shared commodities. Specifically, farmers who live in drier areas with less income are more likely to receive more commodities than they give. We also find that the length of time a household has been established in the area is significantly related to commodity sharing. Further, commodity sharing follows the pattern of harvest and food storage over the course of the year, with households giving the most commodities at times when food storage levels are higher, that is, post-harvest. The study sheds light on the relationship between commodity sharing as a coping mechanism and environmental heterogeneity in a region prone to seasonal food insecurity.

## 1. Introduction

Smallholder agriculture is critical to global food security [1], yet smallholders are highly susceptible to environmental shocks exacerbated by increasing climate variability [2]. Because “ecosystems and the social systems that use and depend on them are inextricably linked” [3 para. 6], to survive in a socio-environmental system increasingly characterized by environmental instability, communities must employ coping strategies to manage such instability. Communities must be able to change in response to shifts in their ecological systems so they can remain functional [3–5]. A considerable amount of research on smallholder adaptive capacity has focused on natural hazards impacts [6]. Additional research has investigated adaptation to climate trends evolving over annual or decadal time periods including drought in small-scale irrigation farming systems [2, 7], the role of traditional agricultural knowledge in the corpus of farmers’ agricultural knowledge [8], barriers to crop diversification [9], and the effect of population pressure on levels of resilience [10].

To mitigate climate shocks, farmers and households engage in multiple strategies on the ground to try to reduce the effects of these shocks, including adjusting seed selection, planting dates, and other cultivation techniques; consuming less or less desirable food; and engaging in piecework employment [11–14]. Such strategies have been identified in the literature as either coping strategies or adaptations. Coping strategies have been characterized as shorter-term, easily reversible, and undertaken ex-post, while adaptations have typically been described as strategies employed in the longer-term, those that are not temporary, and are sometimes able to be undertaken in anticipation of a shock [15].

Because of this ambiguity in terms, Burnham and Ma [13] echo Agrawal’s [16] call for a new classification system for these strategies. In their review paper, Burnham and Ma [13] create a typology of strategies that bypasses competing definitions of adaptation to climate change and groups all behaviors identified in their review as “risk-reducing strategies.” These strategies range from crop diversification to labor migration to stealing, encompassing a range of time periods and scales. Another category of behavior is communal pooling, or the distribution of risk across households, which includes activities like relying on social networks for labor exchange and food [13, 16], and is one of Agrawal’s [16] five categories or types of risk management or risk pooling. Communal pooling of resources has also been demonstrated to contribute to social resilience within homes as well as communities [17]. These pooling activities have the potential to spread household risk across a community by overcoming market failures that many rural African communities face (e.g. lack of access to formal insurance and/or savings markets [18]). In this study, we examine one particular type of communal pooling activity—commodity sharing—as a risk-reducing strategy that Kenyan smallholder farmer households employ in times of climate-related stress. Commodity sharing here is operationalized to include not only commodities given by households, but also commodities received by households.

## 2. Commodity sharing as a risk-reducing strategy

Research across social science disciplines has studied resource sharing or communal pooling as risk-mitigating or adaptive strategies in different ways. In addition to historical and ethnographic accounts that document hazards and risk management in Africa generally [19–21], such as McCabe’s long-term work to understand shifting subsistence strategies of African pastoralists in response to changing economic, environmental, and political conditions [21–22], research into commodity sharing and exchange has addressed the role of food transfers in household coping strategies. Much of this work stems from research on behaviors found in hunter-gatherer societies [23–25] that has identified, for example, reciprocal food sharing as one of the main risk reduction activities undertaken by the Basarwa in northern Botswana [24].

Evolutionary approaches have focused on sharing and exchange in terms of adaptive function or fitness, or to inform debates around human cooperation and sociality, proposing explanations for transfers that range from kin selection to signaling (i.e., a successful quest for food indicates, or signals, valuable characteristics of the food seeker) [26–29]. A cross-cultural examination of four evolutionary models (kin selection, reciprocal altruism, tolerated scrounging, or when food distributed by those having more of the resource to those having less is not contingent on having received food, and costly signaling) across 33 hunter-gatherer groups and 13 forager-agriculturalist groups showed that each model contributes to explaining variance in food sharing behavior, and as such, behavior cannot be generally ascribed to one theory or another [27]. Cultural, political, and social factors provide additional explanations for sharing. For example, the Maasai in Kenya and Tanzania have a particularly strong ethos of solidarity and sharing among community members [30]. For the Maasai, sharing food among community members is part of ritualistic and symbolic dimensions of life [31].

Mauss’ [32] classic work has served as a foundation for understanding commodity sharing in terms of gifts, an approach which continues to be theorized, for example, in terms of utilitarianism (involving rational actors and reciprocity) versus anti-utilitarianism (gifts given freely) of gift-giving [33]. Economic work on risk-sharing, commodity transfers, and consumption smoothing (moving from fluctuations in consumption to a path of stable consumption) in Africa often frames risk in a more general way than specifically in terms of climate change, and identifies instances in which households work to smooth consumption [34–35], or examines insurance relative to shocks [36]. Using data on migration and income transfers, research has found evidence of consumption smoothing among individual households of the same ethnic group in Cote d’Ivoire, and in particular, smoothing for households in geographic regions that were least likely to have access to formal financial arrangements as smoothing mechanism or insurance [34].

More recently, researchers in diverse fields have taken a network approach to examine risk-sharing and commodity exchange [37–43], and recognize the role of social capital in adapting to climate change [44]. Baggio, Kofinas, and colleagues’ [40, 42] research with indigenous communities in Alaska explicitly models the multiple networks within which households are embedded in terms of subsistence goods and services. They identified households who were key to maintaining a robust flow of these goods and services, and found that it is not resource depletion but the breakdown or erosion of social ties that leads to the disruption of subsistence flows.

This paper investigates socio-environmental dynamics related to commodity sharing in smallholder agricultural systems. Previous research on commodity sharing in rural settings, such as that outlined above, has focused on its social, cultural and political dimensions, but little research has explored the interplay between environmental heterogeneity and commodity sharing. When research has focused on environment-related risk and coping and adaptation, it has tended to focus on disaster recovery, broader measures of climate variability and risk, or individuals’ perceptions of risk relative to household behavior. Compared with measures like long-term climate trends, which necessarily miss a lot of local heterogeneity in climate, especially in places like sub-Saharan that suffer from a lack of fine-scale climate data, the environmental heterogeneity we focus on in this paper is a measure of risk that does not rely on individual perceptions of such risk, has clear impacts on households in terms of food and water insecurity, and is more immediately pertinent to those living in the area. To contribute to the understanding of socio-environmental dynamics of smallholder risk-reducing strategies, and to complement the more commonly analyzed social dimensions of commodity sharing, this paper explores the interplay between environmental heterogeneity and social dimensions of coping with risk. Because there is high spatial variability of rainfall across the study area, we are able to investigate commodity sharing in areas with different degrees of reliance on irrigation for crop production. The environmental heterogeneity around Kenya’s “water towers” (areas of higher elevation and higher rainfall that contribute streamflow to and form a regional precipitation gradient with the surrounding drylands), along with variable access to locally-governed piped water for irrigation, present valuable platforms with which to investigate commodity-sharing dynamics under different conditions. This study specifically asks the following questions: Does commodity sharing, measured as net inflow of commodities to a household, fluctuate with this environmental heterogeneity, that is, are there some households who provide more commodities in the study area, and other households who rely more on these commodities, in relation to water availability? What other factors predict commodity sharing?

We consider environmental heterogeneity in terms of a proxy variable and as actual precipitation in the region in two separate analyses. Our conceptualization of net value of commodities shared is inspired by attempts in economics to establish welfare measures that take into account not only production, or in this case, sharing out of commodities, but consumption, or in this case, receiving commodities [45–47]. Here then, net commodity sharing can be thought of as a measure of, in economic terms, “welfare,” or for other fields, well-being. In the context of households coping with shocks, such a measure of well-being serves as an indicator of a household’s potential for coping and adaptation. With the goal of looking at the spectrum of commodity sharing across communities and across a precipitation gradient, and in terms of communal pooling to reduce risk in the community, we believe that it makes sense to look at net as a well-being measure in order to place the phenomenon more appropriately within the context of households receiving and giving commodities. We provide further details on these variables and analyses in section 4.3 below.

## 3. Study area

**Fig 1:**
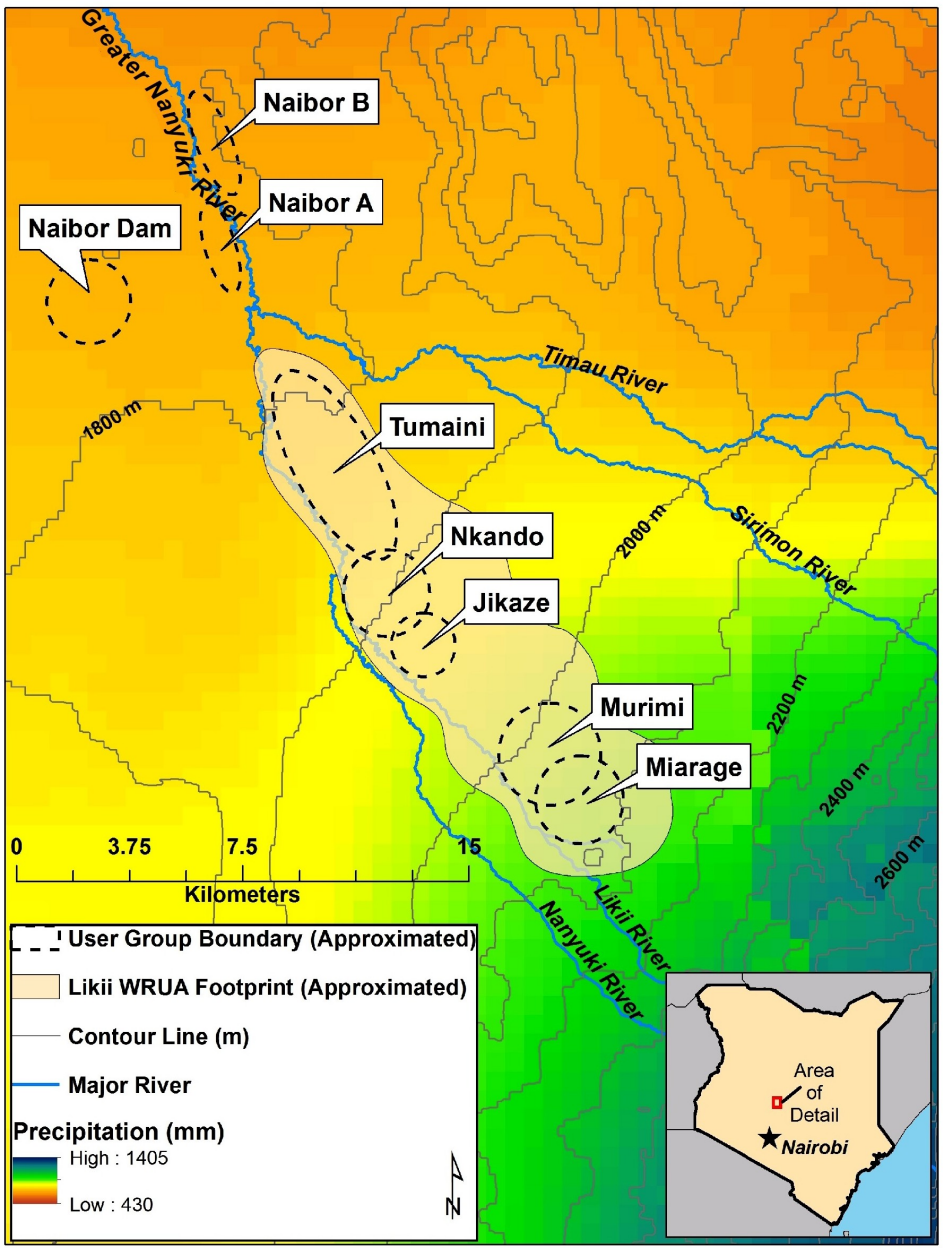
Study site.

The Greater Nanyuki river system is located on the northwestern slopes of Mount Kenya and is made up of multiple tributaries, of which the Likii River generates the greatest average flow (Fig 1). The Likii subcatchment (about 180 km^2^ [48–49]) is typical of a highland-lowland agro-ecosystem. Upstream, the climate is wetter and cooler, with average annual rainfall estimated at approximately 1,400 mm. At the lower reaches, the climate is warmer and drier, and the average annual rainfall drops to less than 450mm. As is the case in the surrounding region, the subcatchment experiences two rainy seasons per year: the long rains, lasting from approximately March to May, and the short rains, lasting from approximately October to December. The region’s climate has exhibited high interannual variability in the timing and magnitude of both the long and short rains over the last three to four decades [50] and a marked reduction in rainfall during the long rains, along with increased temperatures, since the 1960s [51].

The subcatchment is part of the larger upper Ewaso Ng’iro Basin, an area that was primarily occupied by Maasai and Samburu pastoralists up to the early 1900s [52]. Much of this land transitioned to large ranches and farms owned by white settlers during the colonial era, and then, following independence, these areas were subdivided into small plots where farming takes place today. Kikuyu and Meru ethnic groups now primarily populate the humid to semi-arid zones most suitable for sedentary farming—including the Likii subcatchment—and comprise the majority of our study population. In the semi-arid to arid rangelands in the lowland areas further from Mount Kenya, Maasai, Samburu, and other pastoral groups are more prevalent. Throughout the basin, including within the Likii subcatchment, a rapid population increase has taken place, from 50,000 residents in 1960 to 500,000 in 2000 [50]. Much of this growth has been driven by the in-migration of individuals seeking suitable farmland or in pursuit of other economic activities.

**Fig 2:**
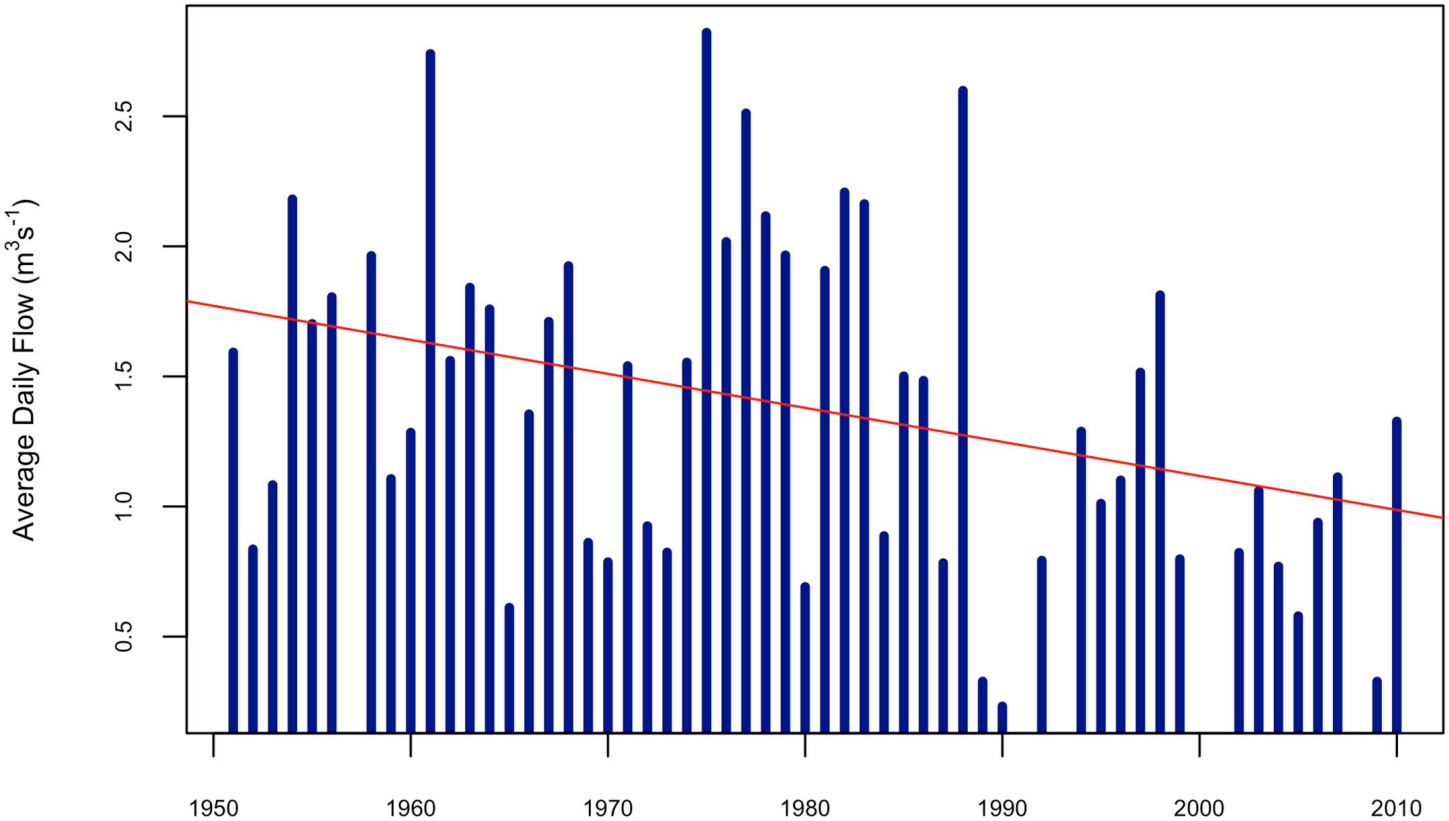
Plot of average daily streamflow in the Likii River from 1950 to 2010 (blue) and associated trend line (red). Years with fewer than 183 observations (half the year) were left blank. Data are drawn from readings of a gauge operated jointly by the Ewaso Ng’iro North Catchment Authority (ENNCA) and the Centre for Training and Integrated Research in ASAL Development (CETRAD) in Kenya.

As illustrated for the Likii River in Fig 2, average daily streamflow in the subcatchment has been decreasing since the 1960s. Previous studies have attributed this decrease to regional population growth and corresponding increases in water abstraction for irrigation and household uses [53–55]. Coupled with this decline, water availability asymmetries produced by the rainfall gradient within the catchment touched off concerns from downstream users regarding water security and catalyzed conflicts between upstream and downstream users. In response, government and non-government officials began encouraging formation of Water Resource Users’ Associations (WRUAs) in the late 1990s as a means to more effectively coordinate among water users and to facilitate communication between stakeholders and regulators [56]. WRUAs are part of a polycentric system of water management, consisting of multiple centers of decision-making, each constructing its own sets of rules and each encouraging public participation in governing the water resource. The Likii WRUA was formed in the late 1990s to coordinate upstream-downstream water use, share information, and resolve disputes among actors [55]. It is made up of individual riparian water users, commercial farmers, one municipality, and communities of water users, known as Community Water Projects (CWPs). One of the primary functions of a WRUA is to facilitate the equitable distribution of surface water to user groups within the same catchment and to increase the potential for downstream users to receive water even in times of water shortages.

In this study, we focus on households within eight communities: five CWPs of the Likii WRUA (Miarage, Murimi, Jikaze, Nkando, Tumaini) and three water user groups along the Greater Nanyuki River (Naibor Dam, Naibor A, Naibor B), downstream of the Likii WRUA (Fig 1). The three downstream groups are user associations that follow river water usage rules set by a manager, but unlike the CWPs within the Likii WRUA, they do not have piped water infrastructure. The five Likii WRUA CWPs are spread linearly along the Likii River and therefore span a portion of the subcatchment’s rainfall gradient.

As members of a CWP, farmers receive water through a series of pipes that divert water from the Likii River and transport it directly to their households, where it is then used for domestic and irrigation purposes. Members are responsible for collectively crafting formal rules that allocate water to member households and sanction members if water is misused. Farm plots within the Likii WRUA CWPs are, on average, two acres in size, and maize, mixed beans, and potatoes are most often cultivated. Average annual household income across all households we surveyed (sample details in section 4 below) in the five Likii WRUA CWPs in 2012 was close to 207,000 Kenyan shillings (approximately 2,500 USD in 2012).

Downstream from the Likii WRUA, the three water user groups comprise smallholder farmers who are primarily Kikuyu and who cultivate plots at two sites located along the Greater Nanyuki River and at a water reservoir constructed near the Naibor Dam (also called the Kiburuti Dam Project), which became operational in 2011 following support from Kenya’s Ministry of Agriculture. The riparian farmers cultivate larger fields than those positioned at the dam. Unlike the CWPs within the Likii WRUA, these farmers withdraw water directly from the Greater Nanyuki River. At the dam, farm plots are typically quite small (< 1 acre), but a variety of crops are grown, including maize, kale, cabbage, and carrots. These smallholders either fetch water directly from the dam using jerry cans or abstract it using one of several foot pumps that are shared among the dam members. Average annual household income across households we surveyed in the three downstream communities in 2012 was 150,000 shillings (approximately 1,740 USD in 2012), about 25% lower than in the upstream CWP communities.

## 4. Methods

### 4.1 Sampling and data collection

Household-level surveys (see S1 Appendix A for instrument) documenting agricultural and water governance practices were conducted from the first week of June through the first week of July 2012. All survey protocols were approved by the Institutional Review Board at Indiana University. We collected information concerning the employment activities of household members, perceptions of water availability, and farmer cultivation decisions. Surveys were conducted by Kenyan enumerators in local languages (primarily Kikuyu, Maa, Embu, and Meru), and respondents provided verbal consent. We did not ask farmers to provide written consent because a subset of respondents did not have sufficient literacy to fully understand written consent language. Household surveys had a duration of approximately 45 minutes and were administered to 315 farming households spread across the eight CWPs or user groups.

Our sampling approach was purposive, intended to sample households who belong to these CWPs or user groups and practice sedentary agriculture, rather than capturing households with pastoral practices (e.g, Maasai or Samburu households). We aimed to sample on average 35 households in each CWP or user group. In one CWP with a much smaller membership than others (39 households), we sampled only 18 households, while in larger CWPs, we sampled over 50 households. In the CWPs in the Likii WRUA, households were sampled along the water distribution infrastructure. Because individual water lines can have different water flow (due to leakage, number of households connected per line, location along the line), we randomly sampled households across each major line to capture diversity in water flow and thereby include households with both abundant and deficient water resources [57–58]. In all eight groups, after we successfully administered a survey at a household, we skipped the next two households before stopping at the third household to conduct the next survey. If available, the head of the household was interviewed. If the head of household was not present, we interviewed the spouse. If neither of these people were available, we skipped that household and went to the next neighboring household.

To obtain information about commodity sharing, respondents were asked to “describe any commodities that you either received as support, or gave to support others during the past year. Commodities include agricultural crops and livestock, among other things.” Commodities do not include labor, land, or tools. Respondents were asked for commodity type and amount, the source or recipient of the commodity, the week and month that the transaction took place, and the commodity’s monetary value. Respondents could list as many commodity transactions as they wished, and were not asked to personally identify the source or recipient; rather, these were named generally as a non-governmental organization (NGO), government, faith-based organization (FBO), school, neighbor, or family member. Respondents were also asked whether each transaction was permanent or something borrowed/lent. 99.4% of all transactions were characterized as permanent, and thus we do not consider this aspect of transactions in any statistical tests below.

### 4.2 Survey descriptive statistics

Of the 315 households interviewed, 181 (57%) reported that they shared commodities. Over fifty different commodities were listed by respondents, with the most frequently listed being maize, beans, cooking oil or fat, soybeans, potatoes, and milk. Table 1 shows the breakdown of the number of households in each CWP or user group, the number of households we surveyed in each community, and the number of households who shared commodities in each community. Table 1, S2 Appendix B shows the commodities shared by all communities, and Table 2, S2 Appendix B shows the breakdown of commodities shared in each of the eight communities.

**Table 1:** The number of households in each CWP or user group, the number of households we surveyed in each community, and the number of households who shared commodities in each community.

CWP/water user group	Number of households (estimated)	Number of surveys conducted	Number of sampled households who shared commodities
Miarage A	125	42	26
Murimi	366	47	30
Jikaze	39	18	9
Nkando	150	41	22
Tumaini	87	51	49
Naibor A	45	19	15
Naibor B	45	24	16
Naibor Dam	30	14	14
Totals	887	315	181

**Table 2:** Descriptive statistics for households who shared commodities (N = 181). At the time of data collection $1 US was worth about 83 Kenyan shillings.

Variable	Range	Mean	Median	Mode	SD
Net value of commodities shared (i.e. commodities received minus commodities given), Kenyan shillings (KSh)	-30,000–13,760	-1,355	-300	-300	4,392
Distance from fixed point upstream, meters	3,070.1–30,273.2	15,543	14,602.6	3,070.1	8,482.7
Household size	1–12	4.8	5	5	1.8
Years lived here	0–52	17.7	17	10	11.2
Household total annual income from all sources (KSh)	2,400–1,428,000	177,672	96,000	20,000	233,505

In Tables 2 and 3 below and Tables 3 and 4, S2 Appendix B, we present additional descriptive statistics for the 181 households that shared commodities. Tables 2 and 3 show demographic variables. Table 3, S2 Appendix B shows the breakdown of whether households gave or received goods, or did both. Then, to assess how much commodity sharing occurred among family and neighbors (the majority of sharing partners) each month, Table 4, S2 Appendix B shows the value of commodities shared (given or received) among family and neighbors as a proportion of all commodities shared each month. In addition to displaying the monetary values, Table 4, S2 Appendix B also presents the percent of sharing transactions occurring among family and neighbors as a proportion of all commodity sharing transactions each month.

**Table 3:** Descriptive statistics for households who shared commodities (N = 181).

Variable	Number of households (%)
Someone in household speaks Kikuyu	138 (76.2%)
Male sex of household head	160 (88.4%)

**Table 4:** Variables in the analysis. At the time of data collection $1 US was worth about 83 Kenyan shillings.

Variable name	Variable description	Range	Mean	Median	Mode	SD
Fixed effects						
Net (dependent variable)	Net value of commodities shared (i.e. commodities received minus commodities given), Kenyan shillings (KSh)	-30,000–5,680	-1292	-300	-300	3,854
Distance to upstream	Distance from fixed point upstream, meters	3,070–30,273	15,207	14,475	5,985	8,691
Kikuyu	Does someone in household speak Kikuyu	0 = no 1 = yes	75.3% speaks Kikuyu	1	1	0.43
HH size	Household size	1–12	4.8	5	5	1.8
Stay	Years lived here	1–52	18	18	22	11.2
Sex of HH head	Sex of household head	0 = Male 1 = Female	12.1% female	0	0	0.33
HH income	Household total annual income from all sources (KSh)	2,400–1,428,000	176,363	94,000	60,000	236,475
Membership	Number of members in community water project	30–366	111	66	45	112
Random effects						
Likii	Member of Likii WRUA or not	0 = no 1 = yes	74.1% members			
CWP code	CWP or user group	Eight groups				

### 4.3 Variables and statistical tests

In accordance with our conceptualization of “sharing” as both the giving and receiving of goods, our outcome variable, “net value of commodities shared,” represents the value of commodities each household reports having received minus the value of commodities each household reports having given. In contrast with other potential measures of sharing (e.g. the absolute value of a household’s sharing transactions, an effective measure for reflecting each household’s level of commodity sharing activity), our measure has the benefit of emphasizing each household’s position as net contributors to or net beneficiaries of the commodity sharing market, and to what extent. Even in the case of households that both contribute and receive, this netted measure of community-level reliance serves as an indicator of a household’s potential for coping with shocks. Further, examining household wellbeing in terms of net flows of commodities relative to others contributes to an understanding of community-level, environmentally-linked household coping, emphasizing that some households are heavily reliant on support from the community, whereas others are contributing more to community support.

First, to determine whether commodity sharing follows a spatial precipitation gradient, measured using a proxy variable for rainfall at the household level, we fit a multilevel mixed-effects model to the data. Second, we look at sharing of commodities over time, using a Pearson correlation test to investigate whether there is a correlation between actual monthly rainfall amounts in the region and the total net value of goods being shared by all households in the communities each month. Combining these two tests first lets us understand the effects of environmental heterogeneity and other social factors on commodity sharing at the household level for a year, while the causation test disaggregates the sharing data to monthly and compares it with rainfall data across the region, providing temporal context and further evidence for the role of environmental factors in commodity sharing.

For the first test, we use a multilevel mixed-effects model due to the presence of both community-level and household-level predictor variables and to account for households being members of CWPs/user groups located either within or outside of the Likii WRUA. Multilevel models allow for analysis of relationships both within and between different levels of grouped data. Linear Mixed Models with Random effects were performed using the SAS MIXED Procedure [59]. The fixed effects are parameterized as in a traditional OLS regression (variables and estimates listed in Tables 4 and 5), while the Random statement in Proc Mixed is used to add a random term to the intercept for each CWP (u_j_), as well as a covariance term for households within CWP. The equation of the model we fit:

**Table 5:** Estimates of fixed effects.

	Unstandardized coefficient (Std. Error)	Standardized variables (Std. Error)
Intercept	-6,008.23 (1648.32)	-.04 (.09)
Distance to upstream	.17*** (.05)	.38*** (.11)
HH income	-.003** (.001)	-.20** (.08)
Membership	7.4 (3.8)	.22 (.11)
Kikuyu spoken	361.12 (704.98)	.09 (.18)
Female HH head	216.44 (922.44)	.06 (.24)
Stay	58.38* (27.65)	.17* (.08)
HH size	112.11 (168.51)	.05 (.08)
Random effects	Covariance parameters (Std. Error)	
Likii (CWP)	52,470 (444,752)	.004 (.030)

*, **, *** denote significance at the .05, .01 and .001 levels respectively. The estimated beta coefficients in Table 5 indicate the predicted change in Net (Y) based on a 1 unit increase in each variable (X), and standardized beta coefficients (and standard errors) are also included to indicate the overall size of the effect.

$$\begin{array}{l} {\text{Y}}_{\text{ij}}=\beta_{0}+\beta_{1}{\text{X}}_{1\text{ij}}+\beta_{2}{\text{X}}_{2\text{ij}}+\beta_{3}{\text{X}}_{3\text{ij}}+\beta_{4}{\text{X}}_{4\text{ij}}+\beta_{5}{\text{X}}_{5\text{ij}}+\beta_{6}{\text{X}}_{6\text{ij}}+\beta_{7}{\text{X}}_{7\text{ij}}+{\text{u}}_{\text{j}}+{\text{e}}_{\text{ij}},\\ {\text{e}}_{\text{ij}}∼\text{N}\left(0,\;\sigma^{2}{}_{\text{e}}\right)\\ {\text{u}}_{\text{j}}∼\text{N}\left(0,\;\sigma^{2}{}_{\text{u}}\right) \end{array}$$

i = household, and j = CWP

Where Y = Net value of commodities shared, X1 = Distance to upstream, X2 = Household income, X3 = Membership, X4 = Kikuyu, X5 = Sex of HH, X6 = Stay, X7 = HH size. Coding for each variable is described in Table 4, and fitted Betas are described in Table 5. We calculated the dependent variable in this model, net sharing (“Net”), based on the monetary value reported by respondents of commodities given and received by the household: commodities that households gave for support were considered outflow, and commodities received were considered inflow. Thus, households who gave more commodities than they received have a negative Net value, and households who received more commodities than they gave have a positive Net value. While this construction of Net does not allow us to discriminate between households who, for example, both gave and received a lot or who both gave and received little (effectively both coming to a Net value of zero or near zero), we are not as concerned here with the amount of the transactions, rather, it is the *balance* of transactions at the household level that is of interest. In addition, we have retained all sharing activities with NGOs/FBOs, the government, or schools, because in constructing Net value using transactions over the course of a year, commodities given or received by these entities still constitute part of the household’s balance of sharing transactions. In addition, it is likely that commodities received by these entities could be redistributed in the community during the year. Finally, we note that the majority of transactions and the value of those transactions are among family and neighbors, as described in Table 4, S2 Appendix B. As noted above, 181 households reported that they shared commodities. Eight households were not members of a CWP or user group, and for seven additional households, we could not calculate a value for Net because the question about commodity value was not answered, leaving 166 households for analysis.

For each household, we control for which of the eight CWPs or user groups the household belongs to (“CWP code”) and whether that group is part of the Likii WRUA (“Likii”) by including random intercepts for each CWP code nested in Likii. These random effects account for the correlation or similarity of households within each CWP or user group. In addition, we account for other variables that have been shown to affect households’ ability to employ risk-reducing strategies [60–62], including sex of household head (“Sex of HH head”), household size (“HH size”), total household income from all sources (“HH income”), and number of years the household has been established there (“Stay”). We expect that female-headed households might receive more than they give, due to having less access to resources than male-headed households, that a larger household size would lead to more receiving than giving due to a larger household having fewer excess resources to give to support others, and that length of time the household has been established might lead to those households receiving more goods because of more stable or well-established social ties. We do not include a parameter for respondents’ group or tribal affiliation, as this can be sensitive information to request in a survey setting. We do, however, include whether the household speaks Kikuyu, the language of the majority group in the area. As a proxy for in-group membership, Kikuyu language may be related to households being able and willing to give or receive more commodities from others in the group who are also Kikuyu speakers.

We account for the number of households belonging to a CWP or user group (“Membership”) because this community-level variable could influence commodity sharing. For instance, access to water for irrigation could be limited by too many households affixed to the CWP pipe infrastructure or by too many individuals taking water directly from a single source. Limited potential for irrigation may then lead to diminished harvests, resulting in greater need for assistance from others.

Finally, we include a variable titled “Distance to upstream” which accounts for the distance of the surveyed household from the most upstream abstraction point (i.e., the abstraction point that provides the Miarage CWP with water, see Fig 1). We include this variable for two reasons. First, fine-scale rainfall data for this region are not available, but due to the orographic effect in the study area, households that are farther west and north-west of the peak of Mount Kenya receive less rainfall. Thus, the Distance to upstream variable captures much of this information concerning the precipitation gradient. Second, in using the Distance to upstream variable, we acknowledge that surface water availability diminishes moving downstream, which is due to water use by upstream actors. The Distance to upstream variable reflects our anticipation that more sharing will be necessary if both sources of water (i.e., precipitation and surface) are in limited supply. We expect that the further downstream a household is located, the more commodities they will receive, that is, the higher their Net value will be. All variables are further described in Table 4. The dataset shared with this paper includes all the variables used in this analysis except for the Distance to upstream variable to prevent human subject identification. We note, however, that our methods have been written in sufficient detail for others to replicate the study in a similar setting.

**Fig 3:**
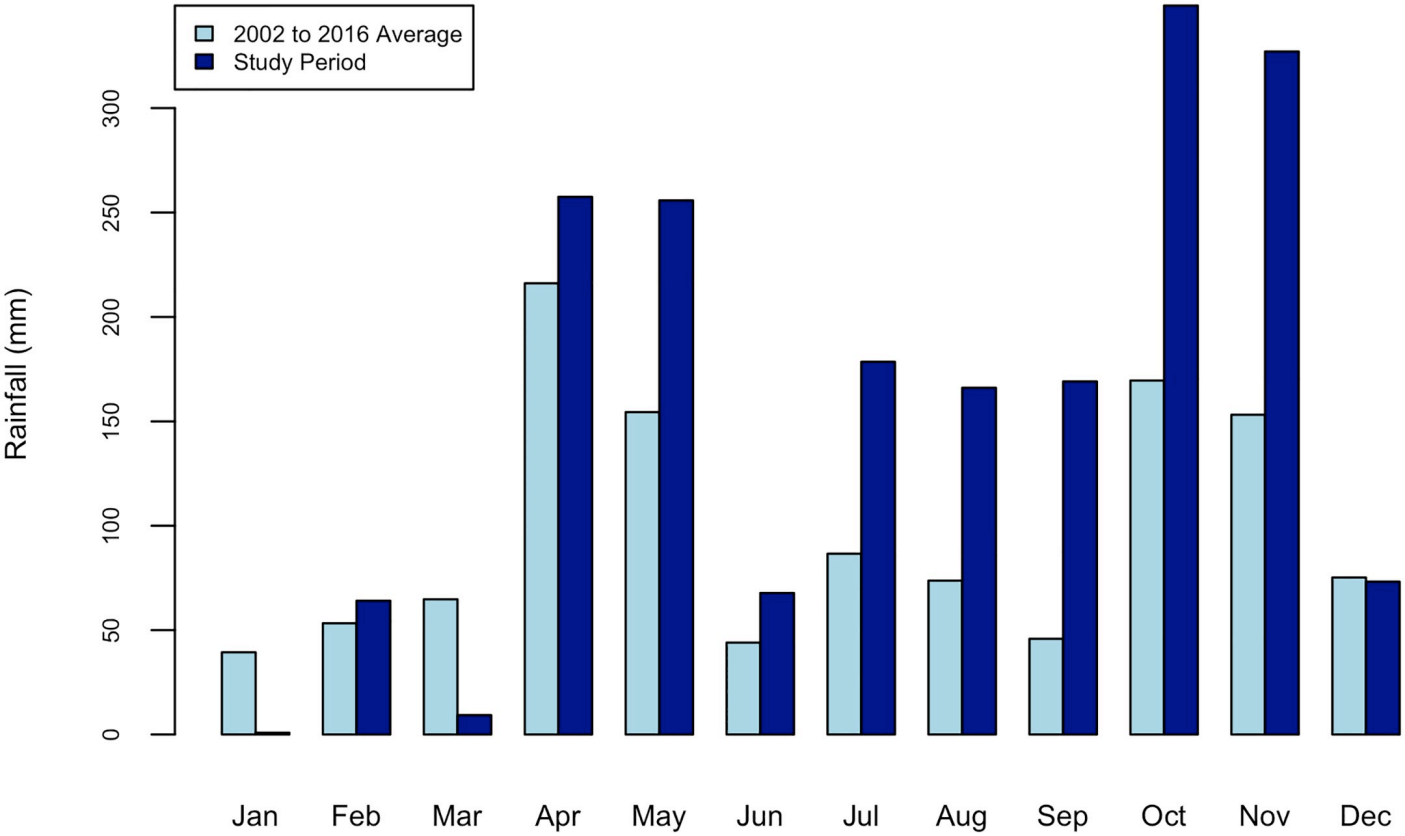
Plot of total monthly rainfall during the study timeframe (August 1, 2011 to July 31, 2012) compared to average monthly rainfall for the 15-year span from 2002 to 2016. Data are drawn from the African Flood and Drought Monitor.

For the second test of whether there is a correlation between the two time series of the monthly rainfall amounts and the total net value of goods being shared by all households in the communities each month, we first differenced each series (x_t_ − x_t-1_) to remove autocorrelation, and performed Pearson correlation on the differenced series. Commodities that survey respondents said were given or received for multiple months, for which they did not remember the month, or for which they did not provide a month were not included in this test. The rainfall variable for this test also characterizes the environmental conditions across the study area. To estimate monthly rainfall and describe those environmental conditions, we downloaded daily satellite-based precipitation estimates [63] for the region through the African Flood and Drought Monitor web page [64] for August 2011 through July 2012. Each daily data set included precipitation estimates for four 0.25° × 0.25° grid cells with total extent 36.90192° E, 37.40192° E, -0.1948268° S, 0.3051732° N, which roughly encompasses the study area. At this latitude, 0.25° × 0.25° grid cells correspond to approximately 28 x 28 square kilometers. These four estimates were then averaged to produce daily mean precipitation values, which we totaled to estimate monthly rainfall values for the study area. To put these totals into context, we compare the 2011–2012 totals with daily precipitation estimates from Drought Monitor data for the 15-year span from 2002 to 2016. Because the gridding scheme changed during the interval between downloads, these data represent a slightly different area, with extent 36.875° E, 37.375° E, -0.125° S, 0.375° N. Largely driven by high rainfall for the months from April to November, total annual rainfall during the 2011–2012 study time frame was much higher than the annual average year over the 15-year span (1,919 mm and 1,176mm, respectively, Fig 3).

## 5. Results

The net value of goods shared follows the precipitation gradient, in that the further downstream a household is located, the greater the value of commodities received (Table 5). When all other variables are held constant, with every one unit (meter) increase in Distance to upstream, Net increases by 0.17 (shillings); therefore, households 1 kilometer apart would have a difference in Net of 170 shillings. Total household income also matters, in that those with more income give more commodities, relative to what they receive (i.e. a lower Net value, or greater net outflow of goods). All other variables held constant, with every one unit (shilling) increase in HH income, Net decreases by 0.003, or for every 10,000 shillings more a household has in income, their Net decreases by 30 shillings. The direction of these relationships suggests that more vulnerable farmers (i.e., those living in drier areas and those with less income) receive higher-value or more commodities, relative to what they give. In addition, we find that as the length of time a household has been established at their current location increases, Net value of commodity sharing increases (i.e. longer residency is associated with more valuable commodities received relative to given). All other variables held constant, with every one unit (year) increase in Stay, Net increases by 58.38. In contrast, our other variables (Membership, Kikuyu language, Sex of HH head, and HH size) are not significantly associated with Net commodity sharing. For variables such as sex of household head, the lack of association may be related to lower variability across the sample.

**Fig 4:**
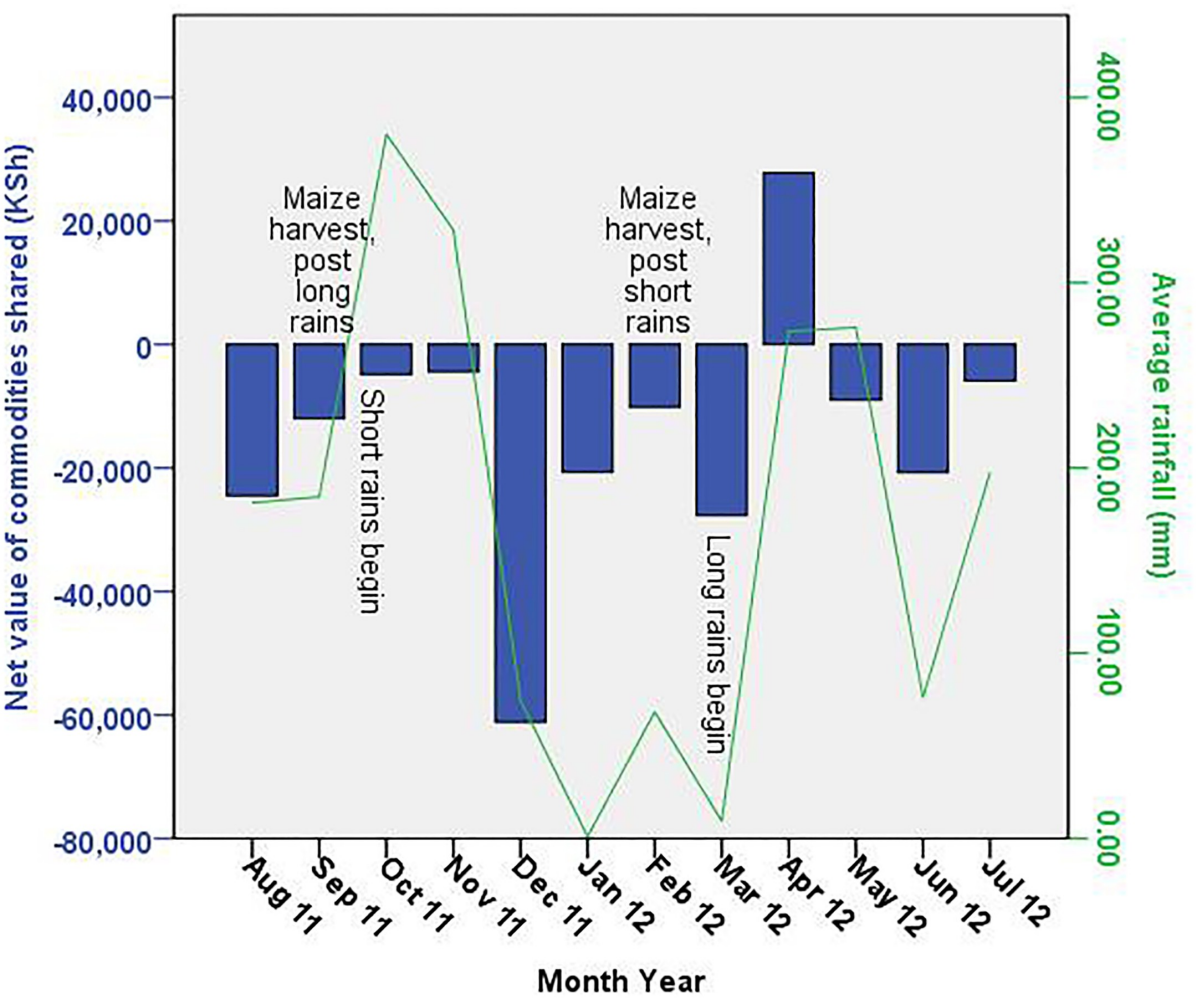
Monthly net sharing for all households (received—given) and monthly rainfall averages, Aug 2011—Jul 2012. Values above the x-axis, i.e., positive values, represent net receiving; values below the x-axis, i.e., negative values, represent net giving.

When we examine the net value of commodities shared over time via the Pearson correlation test, we find that there is a significant relationship (r = .67, p = .025) between rainfall and the net value of goods shared each month. Households give goods in the greatest excess of what they receive (i.e., sharing is the most net negative) when rainfall is at its lowest levels, as indicated by a significant positive correlation between rainfall and the Net value of commodities shared. Fig 4 visually illustrates this trend between monthly rainfall and the net value of goods being shared in these communities.

## 6. Discussion

A pattern emerges across the user group communities in which more commodities, in terms of value, are received than given downstream, where growing conditions are more challenging. Burnham and Ma [13] note that risk-reduction strategies involving communal pooling activities tend to occur in communities where functional social networks have been previously established. Because we have not compared sharing in these communities to sharing in non-water user group communities, we cannot determine whether the water user groups to which households belong have facilitated the formation or strengthening of ties among households. We do note that the majority of commodity sharing, both in terms of the value and number of transactions, occurs among family and neighbors (Table 4, S2 Appendix B). The fact that the length of time a household has been established in a particular location is significantly positively associated with the net value of commodity sharing may indicate that those households who have been in the study area longer have stronger social ties on which they can draw in times of need. But our results offer insight into environmental factors associated with social interactions tied to sharing commodities.

Although distance from upstream predicts higher net value of commodity sharing, we do not conclude that the WRUA should be implicated as ineffective in its charge to help facilitate equal water provision to households. First, the CWPs in the Likii WRUA have their own suite of rules governing water access and their own infrastructural artifacts (e.g. network design and maintenance) that may result in water access or water provision asymmetries. It is possible that each CWP may be taking an equal amount of water from the river, but once water enters the pipes of an individual CWP, it is no longer the WRUA’s responsibility to equitably transport that water to individual households. Second, households are free to decide how to use any water resources they have. For example, some households may favor domestic uses of water, such as for food preparation and cleaning clothes, at the expense of irrigation activities, thereby increasing their dependence on family, neighbors, and/or organizations for food commodities.

In examining the temporal aspect of sharing behavior, we note a few points. April 2012 was an anomalous month with regard to both net sharing trends and the proportion of non-neighborly or family sharing (Table 4, S2 Appendix B). That month’s value appears to reflect an atypical commodity donation from an outside organization. Therefore, we focus our attention on the other months. Households are giving more valuable commodities than they are receiving (i.e. the Net value of commodities shared is negative) for support in months where rainfall is lower (Fig 4). In the study area, total sharing is closest to net neutral at the peak of the long and short rains (Fig 4). Beyond these relationships with precipitation, however, we believe the temporal nature of sharing is best understood through the impact of precipitation on the timing of harvesting and food storage. Maize crop harvesting tends to happen in or before September after the long rains season, and again in January/February after the short rains (though not all farmers plant maize for the short rain season). Following a good harvest, we might expect to see more commodities given to others. Indeed, as depicted in Fig 4, we observe spikes in Net giving in both December and March, perhaps reflecting renewed food storages and increased ability to give commodities to others post-harvest. Thus, we believe that as rainfall affects harvests and food storage, these in turn influence how much and when households share commodities.

The exchange of all commodities measured here does not total net zero, either over the year or within most months (Fig 4). On the whole, households report that they give more than they receive. We note a few possible explanations. First, households sometimes gave commodities to organizations such as schools, the reciprocation of which may not have been captured in our data set if, for example, the school fed the food commodities directly to children. In this case, household giving would be captured within our survey, but household receiving would not. We also did not sample every household within a community, so there are likely transactions for which we captured the giving household but not the receiving household, or vice versa. In addition, there are other communities in the area that we did not survey, so commodities may have been shared with households outside of our study population. For these reasons, as well as the fact that we did not specifically ask for names or households to whom or from whom goods were received, we could not have returned to do random checks on any commodity reporting data. Finally, social desirability bias could have influenced some responses, causing respondents to emphasize giving more than receiving when answering the survey questions, or perhaps individuals simply recall giving commodities more, or more clearly, than receiving them.

As we noted earlier, because our analysis was focused on the balance of transactions, we cannot tell if two households who have near zero Net value both gave and received a lot, or gave and received little. Nor did we examine the quality of commodities shared. Focusing on the transaction level to understand commodity sharing would be a fruitful area for work—for example, thinking about what commodities constitute a short-term consumable (e.g., milk) versus those that could serve as longer-term assets (e.g., a cow) would add important nuance to understanding sharing as a risk-reducing strategy.

Future research would also benefit from the collection of data that would allow us to directly measure reciprocity in commodity sharing, as well as ethnographic data related to cultural and kinship obligations around sharing. Kinship can function as a network for risk-sharing, and within such networks, individuals can be more or less obligated to extend instrumental support or to reciprocate for support received. Such exchange can help individuals cope with shocks, but it can also create constraints such that anticipated obligations can deter individuals from fully undertaking action to protect themselves from risk [65], or in other cases, cooperation among kin may decline overall as people find ways to avoid kinship obligations [66–67]. In this study, while kinship was not a focus, we can see that it matters. Our descriptive data show that, though the proportion of transactions occurring only among family in any month never exceeds about one-third, the value of commodities shared in those transactions sometimes approaches 70% of the value of all commodities shared in a given month (Table 4, S2 Appendix B).

Recognizing that a household’s motivations for engaging in reciprocal sharing are complex—ranging from cultural norms [26, 32] to intentional resilience-building strategies [68–70]—collection of detailed information documenting with and from whom each commodity was shared, and reasons behind sharing, represents an important next step in furthering our understanding of commodity sharing in this socio-environmental context. We would also be interested in comparing sharing behaviors during a high-precipitation year, like that which we observed (Fig 3), with sharing during a lower-precipitation year, when households—particularly downstream households—might be even more likely to face food shortages.

## 7. Conclusion

In this socio-environmental system displaying heterogeneous environmental characteristics, a household’s downstream position, income, and tenure (i.e., length of time living within the community) are significant predictors of commodity sharing. Households that are located further downstream, that is, households that experience less rainfall and have less available water for irrigation, receive more commodities, in terms of monetary value, than other households relative to what they give, and higher-income households give more commodities than they receive. Households share mostly with family and neighbors. Comparing the sharing of commodities to temporal rainfall and agricultural cycles, smallholder households appear to share the most subsequent to the harvest. In other words, net giving is the greatest at times when food storage is likely at levels greater than necessary for short-term household survival. Although we lack the data to definitively place this phenomenon of commodity sharing within the larger contexts of reciprocity or institutional support in the study area, we begin to disentangle the relationship between commodity sharing and environmental heterogeneity in a region that provides crucial, yet variable, water resources to households, and is prone to seasonal food insecurity. Sharing of resources like food or commodities, but also labor, under conditions of resource stress is a “cultural universal,” [71] and we identify paths for future research, such as directly measuring reciprocity and the role of kinship in sharing, focusing on the transaction level and quality of commodities shared, or comparing sharing across years with different precipitation, to clarify and further understand this strategy in the specific context of environmental heterogeneity. While the exact relationship between commodity sharing and environmental factors may vary in different regions where commodity sharing is prominent, our study shows that there is a relationship among these factors, and that it is significant.

## Supporting information

S1 Appendix A Survey instrument. (PDF)

S2 Appendix B Additional tables. (DOCX)

S1 Table (CSV)

S2 Table (CSV)
