# Supplementary material for: Environmental heterogeneity and commodity sharing in smallholder agroecosystems
Source: PLoS One. 2020 Jan 29;15(1):e0228021. doi: 10.1371/journal.pone.0228021 (PMC6988909; doi:10.1371/journal.pone.0228021)
Supplement: S1 Appendix A — (PDF) [file pone.0228021.s001.pdf]

Household questionnare: Snowmelt dependent systems in The United States and Kenya

This survey is being conducted by BLINDED. The focus of this project is on the ability of farmers to adapt to changes in the availability of water in this area. Every precaution will be taken to maintain the complete confidentiality of your responses and personal information. If you choose to participate, you may refuse to answer certain questions, or you may stop participating at any time. Your decision whether or not to participate will not affect your current or future relations with the researchers, BLINDED, or local water associations. Your responses will be summed together with those of roughly 500 other households in Kenya and only general averages from the analysis will be reported. You will not be identified in any published results from this project. This survey should take approximately one hour to complete. Respondents will not receive payment for participating. You indicate your voluntary consent by participating in this interview. If you have questions about your rights as a research participant or have concerns about the study, you may contact the BLINDED.

1. Province: . . . . **PROV** . . . .
2. District: . . . . **DIST** . . . .
3. Location: . . . . **LOC** . . . .
4. Sub-location: . . . . **SLOC**. . . . .
5. Ranch: . . . . **RNCH**. . . . .
6. Group ranch: . . . . **GRNCH** . . . .
7. Village/Locality: . . . . **VILL**. . . . .
8. Household Serial Number: . . . **HHN** . . . .
9. Please list all of the languages that you speak . . . **LANG**
10. Does this household have access to electricity? . . . **ELEC** .
11. How many household members currently reside at this residence? . . . . **HSIZ** .
12. Name of enumerator: . . . . **ENUM** .
13. Date of interview . . . . **DATE**. . Day Month Year
14. Start and end time: . . . . . . **STIME / ETIME**. . Start time End time
15. Name of supervisor: . . . . **SUPER**.
16. Date supervisor checked . . . . **SUPCH** . Day Month Year
17. Name of data entry staff . . . .**DENTRY**
18. Date of data entry . . . . **DENTERED**. . Day Month Year

Section 1: Household attributes

19. Please list and describe the people who live in your household, starting with yourself:

Member age	Gender<br>1=male<br>2=female	Relationship to the respondent (code below)	Education level (code below)	Primary sources of income (code below)	Amount earned annually through these sources
**HH01**	**HH02**	**HH03**	**HH04**	**HH05a, b**	**HH06a, b**
		Self		HH05a:	
				HH05b:	

Relationship to respondent:
1 = head
2 = spouse
3 = own child
4 = step child
5 = parent
6 = brother/sister
7 = nephew/niece
8 = son/daughter-in-law
9 = brother/sister-in-law
10 = parent-in-law
11 = grandmother/grandfather

Education level:
0 = no education
1 = some primary school
2 = completed primary school
3 = some secondary school
4 = completed secondary school
5 = certificate level
6 = some college/university

Income:
1 = on smallholder farm
2 = on commercial farm
3 = in factory
4 = other industrial work
5 = teacher
6 = other civil servant
7 = clerk
8 = shop attendant
9 = non-agricultural piece-work
10 = other (specify)

Income variables:
The first subrow is labeled as variable HH05a, while the second subrow is labeled variable HH05b, as indicated in the first row. HH06 variables follow the same pattern.

List the top two most important sources of income in HH05a and HH05b. List the annual income for these sources in HH06a and HH06b.

Member age	Gender<br>1=male<br>2=female	Relationship to the respondent<br>(code below)	Education level<br>(Code below)	Primary sources of income<br>(code below)	Amount earned annually through these sources
**HH01**	**HH02**	**HH03**	**HH04**	**HH05a, b**	**HH06a, b**
				HH05a:	
				HH05b:	

Relationship to respondent:
1 = head
2 = spouse
3 = own child
4 = step child
5 = parent
6 = brother/sister
7 = nephew/niece
8 = son/daughter-in-law
9 = brother/sister-in-law
10 = parent-in-law
11 = grandmother/grandfather

Education level:
0 = no education
1 = some primary school
2 = completed primary school
3 = some secondary school
4 = completed secondary school
5 = certificate level
6 = some college/university

Income:
1 = on smallholder farm
2 = on commercial farm
3 = in factory
4 = other industrial work
5 = teacher
6 = other civil servant
7 = clerk
8 = shop attendant
9 = non-agricultural piece-work
10 = other (specify)

Income variables:
The first subrow is labeled as variable HH05a, while the second subrow is labeled variable HH05b, as indicated in the first row. HH06 variables follow the same pattern.

List the top two most important sources of income in HH05a and HH05b. List the annual income for these sources in HH06a and HH06b.

20. Are you a widow or a widower? . . . . **WDWR** ☐ Yes ☐ No ☐ No response

21. How old is your oldest child that is still in school? . . . . **SCHL** ☐

22. On a typical day, how many hours per night does that child study? . . . . **STUDY** ☐

23. In the past month, how many times have you or someone in your household had a respiratory illness? . .

ILL . . ☐

24. We would like to know more about your household's sources of energy. Which of the following do you receive energy from?

**Source**	**Variable**	**Yes = 1, No = 0**	**Years in use**
		EN01	EN02
The electric grid [KPLC]	GRID	GRIDEN01:	
A diesel generator	GEN		
Water or hydroelectric system	HYD		
Solar panels	SOL		
Lantern or torch	TOR		
Wood	WOOD		
Kerosene	KER		
Other:	OTR		

Question 24 variables:
The name of each variable in this table is a combination of the prefix from the Variable column, plus the heading from the EN columns. This is demonstrated in the cell labeled GRIDEN01.

Section 2: Land and livestock assets

25. How long have you lived here? . . . .**STAY** ☐ Years

26. In what year did you start farming here? . . . . .**YRFM** ☐

27. Why did you start farming here? **WHYFM**

☐ Farming here is more viable than farming or raising livestock elsewhere

☐ Government policies that encourage settlement in this area

☐ Other: ______

28. If you haven't farmed here your entire life, how did you support yourself or your family before you started farming here? **SPPT**

☐ Farming somewhere else

☐ Raising livestock somewhere else

☐ Other: ______

29. If you haven't farmed here your entire life, are you better off now than you were before you began farming here? **BTROFF**

☐ Yes ☐ No ☐ No response

30. Please explain your answer to the previous question: **BTROFFEX**

______

______

31. How has the total area of land you cultivate changed in the last 5 years? **LNDCHNG**

☐ Greatly increased ☐ Slightly increased ☐ No change ☐ Slightly decreased ☐ Greatly decreased

32. If there has been a change, what is the main reason for this change? **LNDCHNGEX**

______

______

33. How did you originally obtain rights to your land? **LNDRGHT**

☐ Purchased it ☐ Inherited it ☐ Rented it

Rights granted by: ☐ Chief or local authority ☐ Elders ☐ Community ☐ Neighbors

34. Have you had, or are you having, a dispute over your land and your use of it? **LNDCON**

☐ Yes ☐ No ☐ No response

35. Please explain your answer to the previous question: **LNDCONEX**

36. Please describe the livestock owned by members of your household/compound:

Animal	Variable	Number owned	Number sold this year	Total price
		LV01	LV02	LV03
Cattle	CATT	CATTLV01:		
Goats	GOAT			
Sheep	SHP			
Chicken	CHK			
Donkey	DNK			
Other:	OTHR			

37. How has your reliance/dependence on livestock changed in the last 5 years? **LVCHNG**

☐ Greatly increased ☐ Slightly increased ☐ No change ☐ Slightly decreased ☐ Greatly decreased

38. If there has been a change, what is the main reason for this change? **LVCHNGEX**

39. Currently, how important are livestock to the direct support of your livelihood? **LVINCM**

☐ Very Important ☐ Somewhat Important ☐ Not Important

40. Currently, what part of your household income comes from livestock sales? **LVSALE**

☐ None ☐ Some but less than half ☐ Half ☐ Most ☐ All

Question 35 variables:
The name of each variable in this table is a combination of the prefix from the Variable column, plus the heading from the LV columns. This is demonstrated in the cell labeled CATTLV01.

41. Please describe other secondary fields that you use in addition to your primary fields.

Area	Area unit	Village	District	Ranch	Location	Sublocation	Vestment (code below)	Field use (code below)
AF01	AF02	AF03	AF04	AF05	AF06	AF07	AF08	AF09

Vestment/Ownership:
1 = communal
2 = private
3 = state-owned

Field use:
1 = agriculture
2 = livestock
3 = other

Section 3: Agricultural activities

42. How has the amount of land you irrigate changed in the last 5 years? IRRCHNG

☐ Greatly increased ☐ Slightly increased ☐ No change ☐ Slightly decreased ☐ Greatly decreased

43. If there has been a change, what is the main reason for this change? **IRRCHNGEX**

44. What part of your income comes from crops that you irrigate? **IRRINCM**

☐ All ☐ Most ☐ Half ☐ Little ☐ None

45. How much of your water that you use for irrigation comes from rivers and streams? **RVRS**

☐ All ☐ Most ☐ Half ☐ Little ☐ None

46. How much of your water that you use for irrigation comes from boreholes and wells? **WELLS**

☐ All ☐ Most ☐ Half ☐ Little ☐ None

47. Are you a member of an irrigation network? **IRRNTWRK**

☐ Yes ☐ No ☐ No response

48. During the following months of the past year, for how many days was water **available** on your farm (1) through an irrigation network and (2) through a pump? **DAYWTR**

	**2011**								**2012**				
	**May**	**Jun**	**July**	**Aug**	**Sep**	**Oct**	**Nov**	**Dec**	**Jan**	**Feb**	**Mar**	**Apr**	**May**
Through a network													
Through a pump													

49. During the following months of the past year: (1) for how many days did you irrigate your fields, and (2) for how many days was there insufficient water to irrigate your fields? **DAYIRR**

	**2011**								**2012**				
	**May**	**Jun**	**July**	**Aug**	**Sep**	**Oct**	**Nov**	**Dec**	**Jan**	**Feb**	**Mar**	**Apr**	**May**
Days irrigated													
Days with insufficient water													

50. Do you own a water storage tank? **TANK**

☐ Yes ☐ No ☐ No response

51. If yes, how many liters does it hold? . . . . **TANKLTR** ☐

52. If yes, what do you use the storage water for? **TANKUSE**

☐ Irrigation ☐ Livestock ☐ Other: ____________

53. Please sketch the general shape of your farm and identify the fields within the area you manage. For this question, we refer to your "farm" as the area you manage in the area where you are now. We use the term "field" to refer to the areas within your farm that you use for different crops. **GEOSHP**

Sketch

54. Please describe your agricultural activities in the fields you use.

Field ID	Field area		Current crops (code below)	Last year's crop	Percentage of water used	Tillage method (code below)	Irrigation method (code below)	When planted		When harvested		Quantity harvested	Unit of harvest
	Size	Unit						Month	Week	Month	Week		
FLDID	F01	F02	F03	F04	F05	F06	F07	F08	F09	F10	F11	F12	F13

Crops:
0 = fallow
1 = maize
2 = sorghum
3 = rice
4 = millet
5 = wheat
6 = sunflower
7 = groundnuts
8 = soybeans
9 = seed cotton
10 = irish potato
11 = tobacco
12 = mixed beans
13 = bambara nuts
14 = cowpeas
15 = velvet beans
16 = sweet potatoes
17 = cassava
18 = cashew nut
19 = hay
20 = tangerines
21 = oranges
22 = bananas
23 = guavas
24 = paw paws
25 = avocado
26 = water melon
27 = mangoes
28 = cabbage
29 = rapeseed
30 = spinach
31 = tomato
32 = other

Tillage method:
1 = conventional hand hoeing
2 = planting basins
3 = no tillage
4 = ploughing-oxen
5 = ploughing-tractor
6 = ripping
7 = ridging
8 = bunding

Irrigation method:
0 = not irrigated
1 = open unlined ditch
2 = open lined ditch
3 = flooding
4 = sprinkler irrigation
5 = drip irrigation
6 = natural seepage
7 = electrical
8 = gas pump
9 = hand irrigation
10 = treadle Pump/Foot Pump/Moneymaker

Season:
1 = short rains
2 = long rains
3 = dry season

Unit of harvest:
1 = 5kg paper bag
2 = 10 kg paper bag
3 = 50kg sack
4 = 90kg sack
5 = kg (weight)
6 = stacks (hay)
7 = crates
8 = count/number

55. Please describe your use of the crops you have grown this year.

Crop (Code below)	Water used/acre	Seed variety	Seed source	Quantity sold	Quantity unit	Price/unit	Sale method (code below)	Name of sale location	Percent consumed by household for subsistence
**CROP**	**C01**	**C02**	**C03**	**C04**	**C05**	**C06**	**C07**	**C08**	**C09**

Crops:
0 = fallow
1 = maize
2 = sorghum
3 = rice
4 = millet
5 = wheat
6 = sunflower
7 = groundnuts
8 = soybeans
9 = seed cotton
10 = irish potato
11 = tobacco
12 = mixed beans
13 = bambara nuts
14 = cowpeas
15 = velvet beans
16 = sweet potatoes
17 = cassava
18 = cashew nut
19 = hay
20 = tangerines
21 = oranges
22 = bananas
23 = guavas
24 = paw paws
25 = avocado
26 = water melon
27 = mangoes
28 = cabbage
29 = rapeseed
30 = spinach
31 = tomato
32 = other

Sale method:
1 = selling it to a cooperative
2 = selling it on the road
3 = taking it to a local market themselves
4 = taking it to a regional market

56. Please describe the sources from which you receive information about agriculture (e.g., new planting techniques, new tools, new fertilizers, new chemicals, new crops, new hybrids, new marketing options, new crops storage, new credit options, new loan options, creation of new group or society, new learning opportunities…)

**Source**	**Variable**	**A source from which you receive information? 1 = yes 0 = no**	**With what frequency do you receive information from this source?**	**Rank the importance of these sources from 1 to 7**
		SO01	SO02	SO03
Radio	RADIO	RADIOSO01:		
Television	TV			
Newspaper	NWPRP			
Public barazas	BARA			
Local groups	LOCAL			
Informal discussions	INFORM			
Observing other farmers	OBSERV			

57. From whom do you usually receive information about agriculture?

**Source**	**Variable**	**A source from which you receive information? 1 = yes 0 = no**	**With what frequency do you receive information from this source?**	**Rank the importance of these sources from 1 to 13**
		AG01	AG02	AG03
KARI	KARI	KARIAG01:		
CETRAD	CETR			
WRMA	WRMA			
Ministry of agriculture	MINAG			
Ministry of livestock	MINLV			
National irrigation board	NIB			
NGOs	NGO			
Local chief	CHIEF			
Family members	FAM			
Friends	FRND			
Neighbors	NBR			
A group	GROUP			
Private sector	PRVT			
CDF	CDF			
WRUA	WRUA			
Other:	OTHR			

Question 56 variables:
The name of each variable in this table is a combination of the prefix from the Variable column, plus the heading from the SO columns. This is demonstrated in the cell labeled RADIOSO01.

Question 57 variables:
The name of each variable in this table is a combination of the prefix from the Variable column, plus the heading from the AG columns. This is demonstrated in the cell labeled KARIAG01.

58. How many times in the past year have you attended meetings or workshops held by an agricultural extension officer from the government?

. . . . **EXTSN** [ ]

59. If you have met with an agricultural extension officer, how has this affected your activities? **EXTCHNG**

60. Here is a list of actions that people sometimes take as citizens. For each of these, please tell me whether you, personally, have done any of these things during the past year

Activity	Variable	Done activity: Yes = 1, No = 0	Frequency of activity
		ACT01	ACT02
Attended a community meeting	MT		
Contacted a local government councilor	GOV		
Contacted a chief or subchief	CHF		
Contacted a member of parliment	PAR		
Aired grievances	GRVN		

Question 6[variables:
The name of each variable in this table is a combination of the prefix from the Variable column, plus the heading from the ACT columns. This is demonstrated in the cell labeled MTACT01.

61. Please describe (1) any commodities that you received as support, and (2) gave to support others during the past year. Commodities include agricultural crops, livestock, and food from family or neighbors or as aid, among other things.

Commodities received:

Commodity	Permanent = 1 Borrowed = 2	Source (Code below)	Amount	Amount unit	Month received	Week received	Monetary value

Commodities given:

Commodity	Permanent = 1 Borrowed = 2	Recipient (Code below)	Amount	Amount unit	Month given	Week given	Monetary value

Commodities sources/recipients:
1 = family member
2 = neighbor
3 = NGO
4 = faith-based organization
5 = government

Section 4: Rules

62. Please describe the water/irrigation community/systems to which you belong:

Name	Initial cost of membership	Ongoing annual cost of membership	Years of membership

Describe the rules that govern membership to your primary water user association: **MBRULE**

Can be a member:

Can't be a member:

63. Indicate if you agree or disagree with the following sentence: "The membership rules are well implemented." **MBRIMP**

☐ Strongly disagree ☐ Disagree ☐ Neutral ☐ Agree ☐ Strongly agree

64. Please explain your answer: **MBREX**

65. Please describe the rules that govern access to irrigated land in your system: **IRRLNDRUL**

Who can access:

Who can't access:

66. Indicate if you agree or disagree with the following sentence: "The rules in place to govern access to land are well implemented" **IRRLNDIMP**

☐ Strongly disagree ☐ Disagree ☐ Neutral ☐ Agree ☐ Strongly agree

67. Please explain your answer: **IRRLNDIMPEX**

68. Please describe the rules that govern access to water from rivers, streams, or pipes: **WTRRUL**

Who can access:

Who can't access:

69. Indicate if you agree or disagree with the following sentence: "The rules in place to govern access to water from rivers, streams and pipes are well implemented: **WTRIMP**

☐ Strongly disagree ☐ Disagree ☐ Neutral ☐ Agree ☐ Strongly agree

70. Please explain your answer: **WTRIMPEX**

71. If applicable, please describe the rules that govern access to water from wells and boreholes: **GWTRRUL**

Who can access:

Who can't access:

72. Indicate if you agree or disagree with the following sentence: "The rules in place to govern access to water from wells and boreholes are well implemented" **GWTRIMP**

☐ Strongly disagree ☐ Disagree ☐ Neutral ☐ Agree ☐ Strongly agree

73. Please explain your answer: **GWTRIMPEX**

74. In general, how would you describe the extent to which your agricultural practices work well or poorly with the local natural environment? **FIT**
