# Supplementary material for: Environmental heterogeneity and commodity sharing in smallholder agroecosystems
Source: PLoS One. 2020 Jan 29;15(1):e0228021. doi: 10.1371/journal.pone.0228021 (PMC6988909; doi:10.1371/journal.pone.0228021)
Supplement: S2 Appendix B — (DOCX) [file pone.0228021.s002.docx]

**Table 1.** Description of all commodities shared by the 181 households who gave or received any commodities. Households could conduct multiple transactions of different commodities. Households reported a range of 1-8 transactions or sets of transactions (that is, if a household gave a particular commodity to someone every week for multiple months, with no further temporal specificity provided, such instances were counted as a single transaction).

| Commodity shared | Number of transactions in one year | Percent of all transactions |
| --- | --- | --- |
| Maize | 151 | 31.7 |
| Beans | 66 | 13.8 |
| Oil, cooking oil, cooking fat | 40 | 8.4 |
| Soybeans | 28 | 5.9 |
| Milk | 27 | 5.7 |
| Potatoes | 27 | 5.7 |
| Irish potatoes | 21 | 4.4 |
| Peas | 12 | 2.5 |
| Rice | 11 | 2.3 |
| Tomatoes | 9 | 1.9 |
| Chicken | 8 | 1.5 |
| Cabbage | 7 | 1.5 |
| Wheat | 7 | 1.5 |
| Money | 5 | 1 |
| Kale | 4 | 0.8 |
| Kale seedlings | 4 | 0.8 |
| Flour, Maize flour, Napier grass, Porridge flour, Pumpkins | 3 (each) | 0.6 (each) |
| Cabbage seedlings, Carrots, Corn flour, Cow, Fish, Soya mix, Spinach Seedlings | 2 (each) | 0.4 (each) |
| Avocados, Bananas, Butternut, Cowpeas, Goat, Gogget seedlings, Maize seeds, Mixed beans, Onions, Porridge, Salt, Sheep, Sorghum, Soya, Soybean flour, Sugarcane, Sukuma wiki, Sweet potatoes, Trees, Uni mix, Watermelon | 1 (each) | 0.2 (each) |
| Total | 477 |  |

**Table 2.** Breakdown of commodities shared in each of the 8 communities (across 181 households).

|  | CWP or water user project | | | | | | | |
| --- | --- | --- | --- | --- | --- | --- | --- | --- |
| Commodity shared | Miarage A | Murimi | Jikaze | Nkando | Tumaini | Naibor A | Naibor B | Naibor Dam |
| Maize | 21 | 21 | 7 | 15 | 50 | 14 | 13 | 10 |
| Beans | 5 | 8 | 4 | 5 | 19 | 8 | 5 | 12 |
| Oil, cooking oil, cooking fat | 0 | 0 | 0 | 0 | 22 | 7 | 4 | 7 |
| Soybeans | 0 | 0 | 1 | 1 | 17 | 1 | 2 | 6 |
| Milk | 3 | 4 | 3 | 7 | 7 | 3 | 0 | 0 |
| Potatoes | 9 | 9 | 2 | 2 | 3 | 2 | 0 | 0 |
| Irish potatoes | 7 | 6 | 2 | 3 | 3 | 0 | 0 | 0 |
| Peas | 0 | 0 | 0 | 1 | 1 | 6 | 2 | 2 |
| Rice | 0 | 0 | 0 | 0 | 6 | 3 | 2 | 0 |
| Tomatoes | 0 | 0 | 0 | 1 | 5 | 0 | 1 | 2 |
| Cabbage | 2 | 1 | 1 | 0 | 2 | 0 | 1 | 0 |
| Chicken | 2 | 0 | 0 | 3 | 2 | 1 | 0 | 0 |
| Wheat | 0 | 1 | 0 | 1 | 1 | 2 | 2 | 0 |
| Money | 1 | 0 | 0 | 2 | 2 | 0 | 0 | 0 |
| Kale | 0 | 1 | 0 | 2 | 0 | 0 | 0 | 1 |
| Kale seedlings | 0 | 0 | 0 | 0 | 0 | 0 | 0 | 4 |
| Flour, Maize flour, Napier grass, Porridge flour, Pumpkins | 2 | 2 | 0 | 3 | 1 | 5 | 1 | 1 |
| Cabbage seedlings, Carrots, Corn flour, Cow, Fish, Soya mix, Spinach Seedlings | 3 | 0 | 2 | 1 | 2 | 2 | 0 | 4 |
| Avocados, Bananas, Butternut, Cowpeas, Goat, Gogget seedlings, Maize seeds, Mixed beans, Onions, Porridge, Salt, Sheep, Sorghum, Soya, Soybean flour, Sugarcane, Sukuma wiki, Sweet potatoes, Trees, Uni mix, Watermelon | 4 | 1 | 1 | 3 | 2 | 2 | 2 | 6 |

**Table 3.** Breakdown of household sharing activity.

| **Variable** | **Number of households (%)** |
| --- | --- |
| Only gave commodities | 95 (52.5%) |
| Only received commodities | 44 (24.3%) |
| Both gave and received commodities | 42 (23.2) |

**Table 4.** The value of commodities shared (given or received) and percent of sharing transactions among family and neighbors as a proportion of all commodities shared each month, with monthly total rainfall amounts. At the time of data collection $1 US was worth about 83 Kenyan shillings.

| Month Year | Total rainfall (mm) | Number of commodity sharing transactions | Absolute value of commodities shared (KSh) | Percent of commodity sharing transactions among family and neighbors | Percent of commodity sharing transactions occurring among family only | Percent of value of commodities shared among family and neighbors | Percent of value of commodities shared among family only |
| --- | --- | --- | --- | --- | --- | --- | --- |
| Aug 2011 | 181 | 29 | 32,780 | 41.4 | 13.8 | 60.2 | 31.7 |
| Sep 2011 | 184 | 8 | 12,650 | 50 | 0 | 97.5 | 0 |
| Oct 2011 | 380 | 9 | 12,800 | 77.8 | 33.3 | 66.9 | 41.9 |
| Nov 2011 | 328 | 25 | 15,355 | 68 | 20 | 84.5 | 60.8 |
| Dec 2011 | 75 | 28 | 68,030 | 82.1 | 21.4 | 90.9 | 69.2 |
| Jan 2012 | 1 | 33 | 24,825 | 75.8 | 3 | 64.6 | 3 |
| Feb 2012 | 68 | 25 | 19,565 | 60 | 8 | 75.9 | 28.1 |
| Mar 2012 | 9 | 38 | 46,600 | 50 | 2.6 | 41.5 | 3.2 |
| Apr 2012 | 274 | 96 | 41,665 | 26 | 6.3 | 17.18 | 4.2 |
| May 2012 | 276 | 46 | 39,495 | 65.2 | 6.5 | 67.6 | 9.1 |
| Jun 2012 | 76 | 39 | 29,640 | 76.9 | 10.3 | 80.4 | 14.4 |
| Jul 2012 | 198 | 6 | 6,380 | 66.7 | 16.7 | 90.1 | 62.7 |
